# RETRACTION: Wound Healing Rates and Wound Problems of Conventional Circumcision Compared With Ring Circumcision: A Meta‐Analysis

**DOI:** 10.1111/iwj.70204

**Published:** 2025-02-02

## Abstract

**RETRACTION:** WangD. , LiZ. , ChenX. , and WangH. , “Wound Healing Rates and Wound Problems of Conventional Circumcision Compared With Ring Circumcision: A Meta‐Analysis,” International Wound Journal 20, no. 9 (2023): 3699–3707, 10.1111/iwj.14262.37303303 PMC10588352

The above article, published online on 11 June 2023, in Wiley Online Library (http://onlinelibrary.wiley.com/), has been retracted by agreement between the journal Editor in Chief, Professor Keith Harding; and John Wiley & Sons Ltd. Following an investigation by the publisher, all parties have concluded that this article was accepted solely on the basis of a compromised peer review process. The editors have therefore decided to retract the article. The authors did not respond to our notice regarding the retraction.

**RETRACTION:** D. Wang , Z. Li , X. Chen , and H. Wang , “Wound Healing Rates and Wound Problems of Conventional Circumcision Compared With Ring Circumcision: A Meta‐Analysis,” International Wound Journal 20, no. 9 (2023): 3699–3707, 10.1111/iwj.14262.37303303 PMC10588352

The above article, published online on 11 June 2023, in Wiley Online Library (http://onlinelibrary.wiley.com/), has been retracted by agreement between the journal Editor in Chief, Professor Keith Harding; and John Wiley & Sons Ltd. Following an investigation by the publisher, all parties have concluded that this article was accepted solely on the basis of a compromised peer review process. The editors have therefore decided to retract the article. The authors did not respond to our notice regarding the retraction.

